# Peripheral blood CD19+B cell proportion at baseline predicts early complete response to belimumab treatment in patients with refractory lupus nephritis

**DOI:** 10.1136/lupus-2026-002100

**Published:** 2026-08-03

**Authors:** Xinxin Xie, Yan Zhou, Quangang Zhu, Yun Liao, Zhongjian Chen, Weihua Song

**Affiliations:** 1 Shanghai Skin Disease Hospital, School of Medicine, Tongji University, Shanghai, China; 2 Clinical Pharmacy, Shanghai General Hospital, Shanghai Jiao Tong University School of Medicine, Shanghai, China

**Keywords:** Lupus Nephritis, B-Lymphocytes, Lupus Erythematosus, Systemic, Autoimmune Diseases

## Abstract

**Objective:**

Belimumab has been proven to be effective for treating refractory lupus nephritis. However, the meaningful renal response is still far from satisfactory. This study sought to identify immunological biomarkers to stratify patients who are likely to benefit from belimumab therapy.

**Methods:**

We retrospectively reviewed the medical records of patients with refractory lupus nephritis who received 6 months belimumab treatment. Complete response (CR), partial response (PR) and no response (NR) were evaluated, and the predictors of CR were identified.

**Results:**

A total of 28 patients with refractory LN were enrolled. The proportions of CR, PR and NR at 6 months were 39.29%, 32.14% and 28.57%, respectively. The baseline CD19+B cell proportions were significantly higher in CR patients than those in non-CR patients (19.30% vs 10.72%, p<0.001). In the binary logistic regression, baseline CD19+B cell proportion was identified as a predictor of CR in both univariate analysis (OR 1.359, 95% CI 1.098 to 1.683, p=0.005) and exploratory multivariate analysis adjusting for 24-hour urinary protein (OR 1.349, 95% CI 1.092 to 1.665, p=0.005). Receiver operating characteristic showed that the cut-off level of CD19+B cell proportion was 15.12%, with a sensitivity of 90.90%, specificity of 88.20% and an area under curve of 0.888 (95% CI 0.759 to 1.000, p=0.001).

**Conclusion:**

Findings support the baseline CD19+B cell proportion could be used to predict CR to treatment with belimumab in refractory lupus nephritis patients.

**WHAT IS ALREADY KNOWN ON THIS TOPIC**

- B cell-targeted therapy, specifically belimumab, is a solid option for the treatment of refractory lupus nephritis (LN).

**WHAT THIS STUDY ADDS**

- In patients with refractory LN, a baseline CD19+B cell proportion exceeding 15.12% was associated with a significantly increased probability of achieving complete response at 6 months of belimumab therapy.

**HOW THIS STUDY MIGHT AFFECT RESEARCH, PRACTICE OR POLICY**

- Baseline proportion of CD19+B cells may serve as a potential and non-invasive biomarker to predict treatment response to belimumab therapy among refractory LN patients, which needs prospective validation.

## Introduction

 Lupus nephritis (LN) is a common and severe complication of systemic lupus erythematosus (SLE), affecting up to 60% of patients with SLE.^1^ Despite aggressive immunosuppressive treatment, a significant proportion of LN does not respond to treatment and is diagnosed with refractory LN, and eventually progresses to end-stage kidney disease.^2^ Managing refractory LN, therefore, remains a great challenge, in part because its definition remains inconsistent across guidelines. The American College of Rheumatology (ACR) defines it as disease worsening at 3 months or treatment failure at 6 months, whereas the European League Against Rheumatism/European Renal Association-European Dialysis and Transplantation Association (EULAR/ERA-EDTA) criteria define it as failure to achieve PR after 6–12 months of adequate treatment.^3^ The ACR definition enables earlier identification of refractory disease, allowing for timely adjustment of treatment strategy and thereby increasing the chance of achieving early complete response (CR) and improving long-term renal outcomes.

B cells play a pivotal role in the pathogenesis of LN and represent an attractive therapeutic target.^4 5^ Belimumab, a biological agent targeting B-lymphocyte stimulator (BLyS), blocks BLyS binding to its receptor on B cells, which inhibits B cell survival.^6^ Previous studies demonstrated that the addition of belimumab to standard therapy improved the renal response of patients, with no significant difference in the incidence of adverse events.^7 8^ Recent real-world observational studies have also demonstrated its efficacy on refractory LN when added to standard treatment regimens or combined with rituximab.^9 10^ Nevertheless, belimumab is not effective for all patients, and the renal response rate is approximately 50% for the refractory LN. Therefore, appropriate biomarker to identify patients who are likely to benefit from belimumab therapy may guide treatment decisions.

Previous studies have shown that the traditional clinical factors such as proteinuria, serum creatinine and the combination of anti-SSA/Ro60 and anti-dsDNA serotype can serve as predictors of belimumab treatment in patients with LN.^11 12^ However, their predictive value is poor because these predictors are not pathogenetic factors. In the current study, we assessed the efficacy of belimumab in treating patients with refractory LN as defined by the ACR criteria. Additionally, we aimed to find novel indicators that can predict clinical response at the sixth month after belimumab treatment in patients with refractory LN.

## Methods

### Study design and population

**Figure 1:**
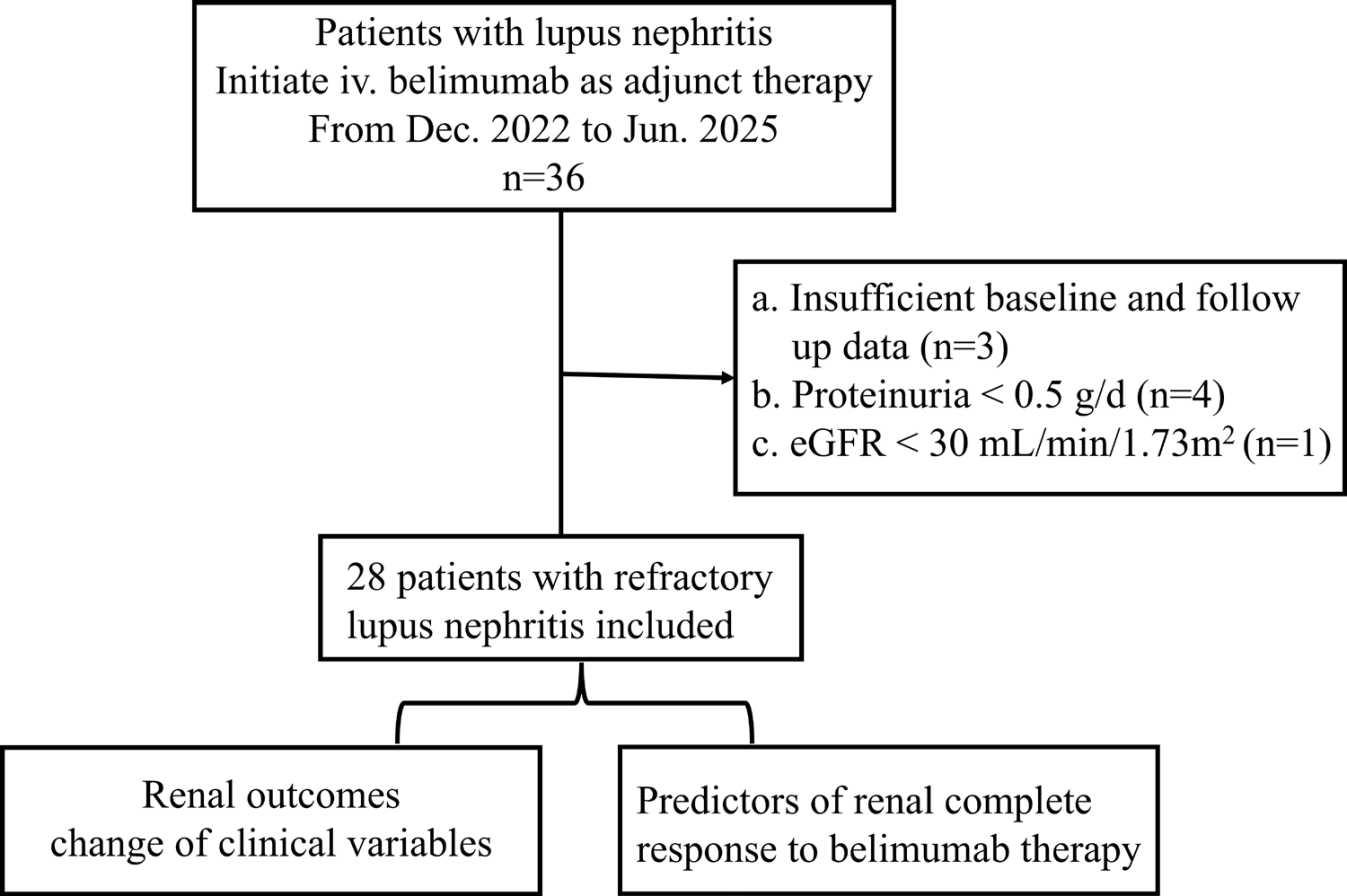
Flowchart of the study. eGFR, estimated glomerular filtration rate.

This was a single-centre retrospective observational cohort study. We enrolled patients from Shanghai General Hospital, Shanghai Jiao Tong University School of Medicine, who had received belimumab for at least 6 months between December 2022 and June 2025. Eligible patients were adults (≥18 years) who met the 2019 EULAR/ACR SLE classification criteria and had refractory LN as defined by the ACR criteria, namely, 24-hour urinary protein ≥0.5 g/day and failure to respond to the induction therapy with mycophenolate mofetil, cyclophosphamide, calcineurin inhibitors or multitarget treatment for at least 6 months. Exclusion criteria included a follow-up period of less than 6 months, active infectious diseases, estimated glomerular filtration rate (eGFR) <30 mL/min/1.73 m^2^ or ongoing dialysis. Based on these criteria, a total of 36 patients were initially screened. Of these, eight patients were excluded: three due to insufficient baseline and follow-up data, four due to proteinuria <0.5 g/day, one due to eGFR <30 mL/min/1.73 m^2^. Ultimately, 28 refractory LN patients were included. The study flowchart is presented in figure 1. The study was reviewed and approved by the Ethics Committee of the Shanghai General Hospital, Shanghai Jiao Tong University School of Medicine (No. 2026029).

### Data collection

Baseline and follow-up data were recorded from the beginning of belimumab therapy. We retrospectively reviewed the electronic medical records of patients for demographic features and clinical information. The clinical information included serum creatinine level, eGFR, serum albumin level, antinuclear antibodies, anti-double-stranded DNA (anti-dsDNA) antibodies, 24-hour urinary protein, C3 and C4 levels, counts and percentage of CD19+B cells, CD3+T cells, CD4+ T cells, CD8+ T cells, and NK cells, as well as the SLE Disease Activity Index 2000 (SLEDAI-2K) score. To address information bias, clinical data were retrospectively extracted by two independent researchers using a case report form.

### Treatment and evaluation of outcomes

All refractory LN patients were administered 10 mg/kg intravenous infusion of belimumab every 2 weeks for the first three doses and every month thereafter. The standard therapy was permitted, including corticosteroids or immunosuppressants. The response to treatment was assessed at 1, 3 and 6 months after induction therapy according to the EULAR/ERA-EDTA definition. CR was defined as proteinuria less than 0.5 g/24 hours and normal or near normal (within 10% change of eGFR if previously abnormal) renal function. Partial response (PR) was defined as an over 50% reduction of proteinuria from baseline and normal or stable eGFR. NR is defined as not achieving CR or PR.

### Statistical analysis

All statistical analyses were performed in SPSS V.25.0 (SPSS, Chicago). Descriptive statistics are presented as mean with SD or median with IQRs for normally distributed and non-normally distributed data, respectively. The independent samples t test was used for comparing the difference between two groups if the data fitted a normal distribution, and if not the Mann-Whitney U test was used. To identify factors associated with CR at 6 months, univariate binary logistic regression was performed, and an exploratory multivariate logistic regression analysis adjusting for 24-hour urinary protein was also conducted. Receiver operating characteristic (ROC) curve analysis was performed to evaluate the predictive value of baseline CD19+B cell proportion for CR at 6 months. The optimal cut-off value was determined using the Youden index. Sensitivity, specificity and AUC with 95% CI were reported. For internal evaluation of the stability of the predictive model, 0.632 bootstrap resampling with 500 iterations was performed. P<0.05 was regarded as statistically significant.

## Results

### Patient characteristics

A total of 28 refractory LN patients who received belimumab treatment were included in this study and followed up for 6 months. The demographic and clinical characteristics of the study population are presented in table 1. The mean age of the patients was 41.18±16.85 years, and 85.71% were female. A total of 24 out of 28 patients underwent a kidney biopsy at diagnosis, their biopsies were class IV (13 cases), class III/IV+V (9 cases) and class V (2 cases). At the start of belimumab therapy, all patients were in active stage of LN. The median amount of 24-hour urine protein and serum creatinine were 2.49 (1.75 to 4.45) g and 60.45 (49.15 to 84.40) μmol/L, respectively. The mean eGFR and SLEDAI-2K were 101.12±41.24 mL/min/1.73 m^2^ and 14.21±5.21, respectively. Anti-dsDNA antibody positivity was detected in 82.14% patients, while hypocomplementemia (defined as C3<0.9 g/L or C4<0.1 g/L) was observed in 75.00% patients.

**Table 1:** Baseline characteristics of the study population

Characteristics	Total (n=28)
Female, n (%)	24 (85.71)
Age (years)	41.18±16.85
Anti-dsDNA antibody positivity, *n* (%)	23 (82.14)
Serum creatinine (μmol/L)	60.45 (49.15, 84.40)
eGFR (mL/min/1.73 m^2^)	101.12±41.24
Urinary protein (g/24 hours)	2.49 (1.75, 4.45)
Serum albumin (g/L)	29.40±6.02
Complement C3 (g/L)	0.67 (0.54, 0.98)
Low C3, n (%)	21 (75.00)
Complement C4 (g/L)	0.14 (0.06, 0.21)
Low C4, n (%)	16 (57.14)
SLEDAI-2K score	14.21±5.21
Count of CD19+B cells (/μL)	154.50 (100.50, 278.00)
Proportion of CD19+B cells (%)	12.03 (10.09, 20.31)
Renal pathology	
IV	13 (54.17)
III/IV+V	9 (37.50)
V	2 (8.33)
Medications	
PSL equivalent (mg/day)	28.57±16.32
HCQ, n (%)	24 (85.71)
MMF, n (%)	23 (82.14)
CTX, n (%)	5 (17.86)

CTX, cyclophosphamide; eGFR, estimated glomerular filtration rate; HCQ, hydroxychloroquine; MMF, mycophenolate mofetil; PSL, prednisolone; SLEDAI-2K, Systemic Lupus Erythematosus Disease Activity Index 2000.

As for concomitant medications, all patients initiated belimumab treatment combined with conventional therapy. The average dose of prednisone (or equivalent) was 28.57±16.32 mg, and 24 (85.71%) patients were prescribed hydroxychloroquine. Regarding immunosuppressive agents, 23 cases (82.14%) received mycophenolate mofetil, and cyclophosphamide in 5 cases (17.86%).

### Therapeutic responses

**Figure 2:**
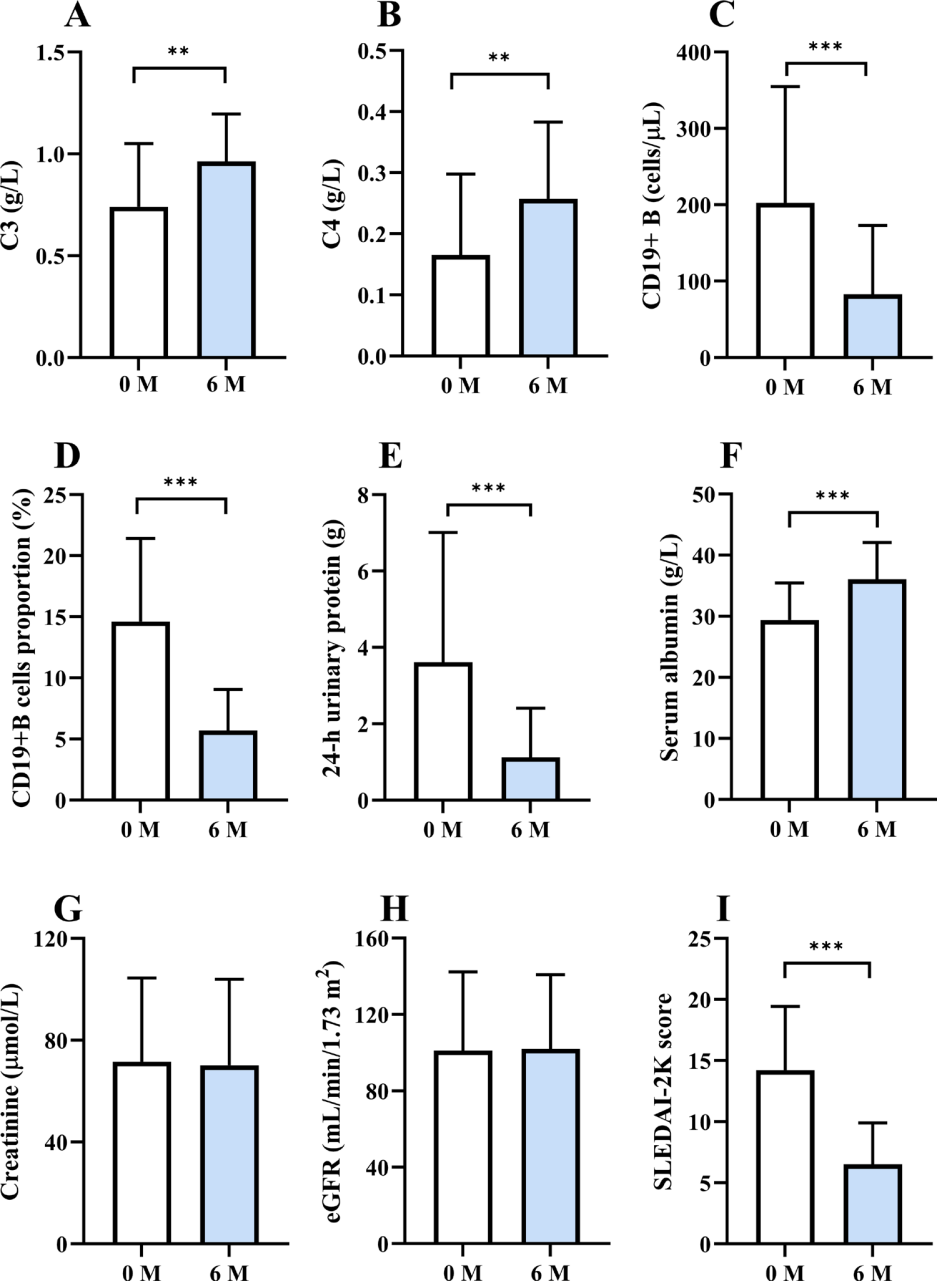
Changes of LN clinical parameters after belimumab treatment. (**A**) C3, (**B**) C4, (**C**) CD19+B cells count, (**D**) CD19+B cells proportion, (**E**) 24 hours urinary protein, (**F**) serum albumin, (**G**) creatinine, (**H**) eGFR and (**I**) SLEDAI-2K score. ^*^p<0.05; ^**^p<0.01; ^***^p<0.001. eGFR, estimated glomerular filtration rate; m, month; SLEDAI-2K, Systemic Lupus Erythematosus Disease Activity Index 2000.

At the endpoint, the conditions of the immune system had improved, manifested in serum C3, C4, CD19+B and CD3+T cells. As depicted in figure 2A,B, the levels of C3 and C4 increased significantly after belimumab treatment, rising from 0.67 (0.54 to 0.98) g/L to 0.90 (0.80 to 1.07) g/L (p=0.001), 0.14 (0.06 to 0.21) g/L to 0.21 (0.17 to 0.38) g/L (p=0.003), respectively. The median count and proportion of CD19+B cells were significantly decreased compared with baseline values, decreasing from 154.50 (100.50, 278.00)/μL to 65.50 (46.50 to 76.75)/μL (p<0.001), 12.03 (10.09 to 20.31) to 4.58 (3.59 to 8.16) (p<0.001), respectively (figure 2C,D). Regarding the effects on CD3+T cells change after belimumab treatment, the count of CD3+T cells has no significant differences (p=0.731). However, the proportion of CD3+T cells was significantly increased (p=0.006) (online supplemental figures S1 and S2). No significant changes were observed in the counts or proportions of CD4+T cells, CD8+T cells or NK cells after treatment (p>0.05) (online supplemental table S1).

Regarding the effects on renal response, it was observed that 24 hours urinary protein showed a significant decrease in the sixth month compared with baseline (0.70 (0.25 to 1.29) g vs 2.49 (1.75 to 4.45) g, p<0.001) (figure 2E). Concurrently, serum albumin levels increased accordingly, with a significant improvement at 6 months compared with baseline (36.07±5.97 g/L vs 29.40±6.02 g/L, p<0.001) (figure 2F). There were no significant changes in serum creatinine (p=0.838) and eGFR (p=0.927) before and after treatment (figure 2G,H).

In assessing the overall improvement of LN, we observed a significant reduction in disease activity according to the SLEDAI-2K scores before and after the belimumab treatment, namely 14.21±5.21 vs 6.50±3.42, respectively (p<0.001) (figure 2I). The improvement of extra-renal manifestations is presented in online supplemental table S2.

### Comparisons between CR group and no-CR group

A total of 11 (39.29%) patients achieved CR to therapy, while nine (32.14%) patients achieved PR and eight (28.57%) patients showed NR. The patients with PR or NR were grouped into the no-CR group for subsequent analysis. The baseline characteristics of the CR and no-CR groups are shown in table 2. Among demographic characteristics and laboratory parameters, there were no significant differences between patients with CR and without it, except that the median proportion of CD19+B cells was significantly higher in the CR group than in the no-CR group (19.30% vs 10.72%, p<0.001). The induction regimen did not appear to be correlated with CR in this cohort.

**Table 2:** Comparison of baseline characteristics between CR and no-CR at 6 months

Characteristics	CR (n=11)	no-CR (n=17)	P value
Female, n (%)	9 (81.82)	15 (88.24)	0.639
Age (years)	36.91±18.54	43.94±15.61	0.289
Anti-dsDNA antibody positivity, n (%)	10 (90.91)	13 (76.47)	0.312
Serum creatinine (μmol/L)	61.10 (44.30, 94.90)	59.80 (45.95, 100.85)	0.817
eGFR (mL/min/1.73 m^2^)	117.42 (67.12, 143.45)	100.11 (59.67, 127.48)	0.430
Urinary protein (g/24 hours)	2.77 (1.69, 4.44)	2.32 (1.44, 4.67)	0.926
Serum albumin (g/L)	30.79±5.19	28.49±6.48	0.334
Complement C3 (g/L)	0.60 (0.33, 0.76)	0.74 (0.58, 1.08)	0.244
Complement C4 (g/L)	0.09 (0.05, 0.26)	0.14 (0.08, 0.21)	0.677
SLEDAI-2K score	15.45±5.66	13.41±4.90	0.320
Proportion of CD19+B cells (%)	19.30 (16.22, 22.74)	10.72 (8.30, 12.03)	<0.001
Proportion of CD3+T cells (%)	75.89 (72.09, 79.57)	83.95 (74.33, 87.03)	0.082
PSL equivalent (mg/day)	25.00 (20.00, 40.00)	30.00 (7.50, 40.00)	0.963
MMF, n (%)	9 (81.82)	14 (82.35)	0.971

CR, complete response; eGFR, estimated glomerular filtration rate; MMF, mycophenolate mofetil; PSL, prednisolone; SLEDAI-2K, Systemic Lupus Erythematosus Disease Activity Index 2000.

### Baseline proportion of CD19+B cell predict CR

**Figure 3:**
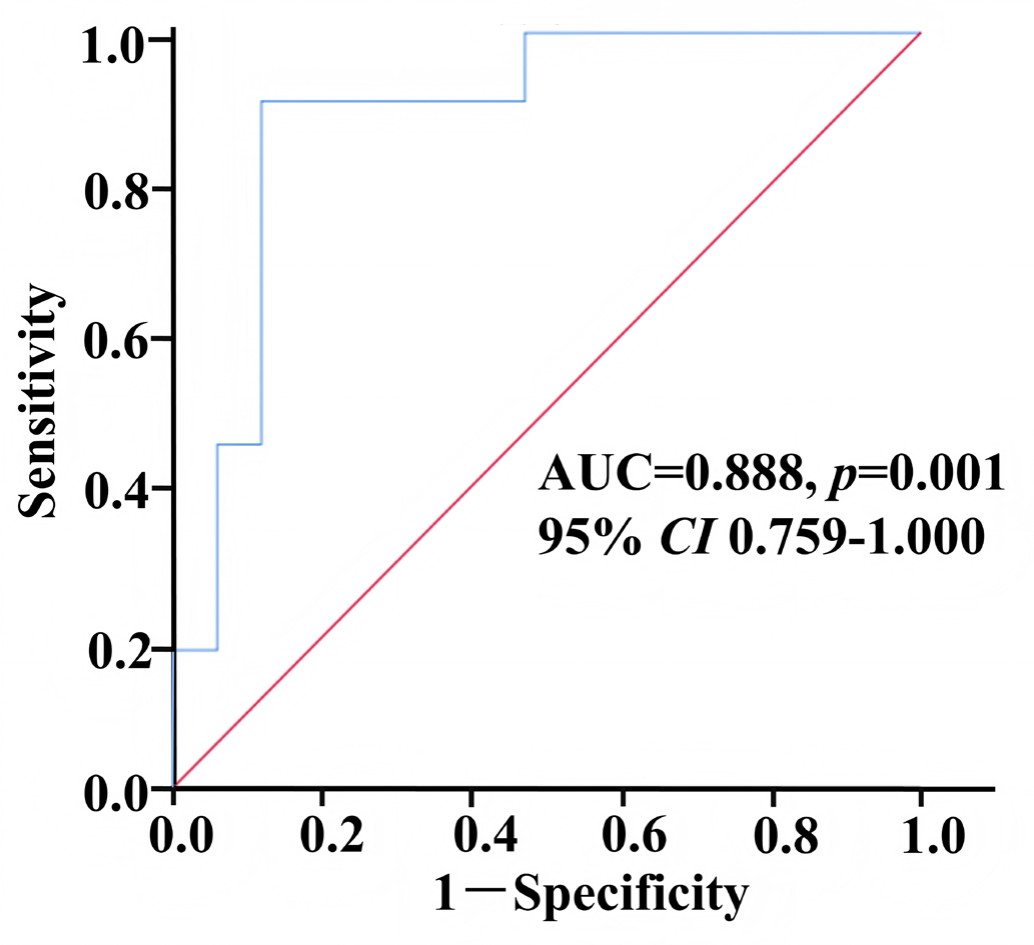
ROC curve analysis was performed to predict CR at 6 months after belimumab therapy. AUC, area under the ROC curve; CR, complete response; ROC, receiver operating characteristic.

**Figure 4:**
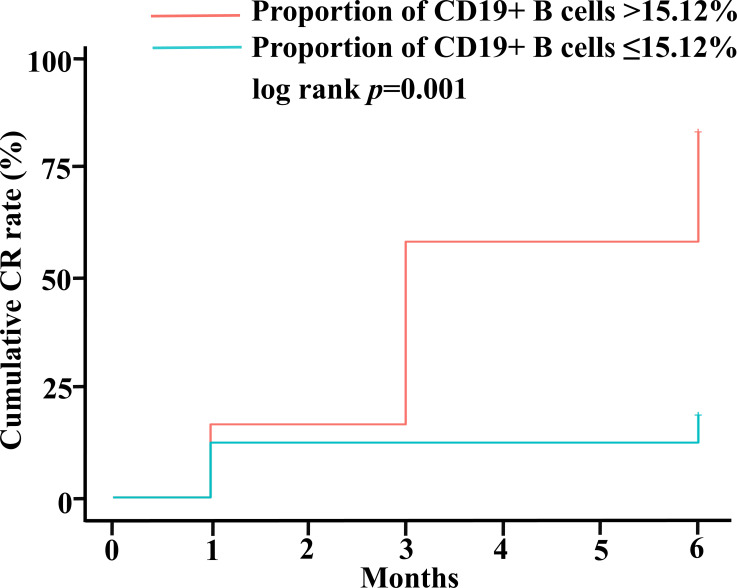
Kaplan-Meier curves of patients with CR according to baseline CD19+B cells proportions. CR, complete response.

To identify factors associated with CR at 6 months, univariate binary logistic regression was performed. As shown in table 3, the baseline proportion of CD19+B cells was significantly associated with CR (OR 1.359, 95% CI 1.098 to 1.683, p=0.005). In an exploratory multivariate logistic regression analysis adjusting for 24-hour urinary protein, baseline CD19+B cell proportion remained a significant predictor of CR (OR 1.349, 95% CI 1.092 to 1.665, p=0.005) (online supplemental table S3). Subsequently, ROC analysis was performed to determine the optimal cut-off value for predicting CR using the Youden index. A cut-off value of 15.12% for the baseline proportion of CD19+B cells was established, demonstrating a sensitivity of 90.9%, specificity of 88.2% and an AUC of 0.888 (95% CI 0.759 to 1.000, p=0.001) (figure 3). To assess the robustness of this predictive model, we performed 0.632 bootstrap internal validation. The bootstrap-corrected AUC was 0.887 (95% CI 0.835 to 0.924), indicating stable predictive performance with a substantially narrowed CI compared with the original analysis (online supplemental table S4). Kaplan-Meier analysis showed that patients with a baseline CD19+B cell proportion above this cut-off had a significantly higher probability of achieving CR (p=0.001, by log-rank test) (figure 4 and online supplemental table S5).

**Table 3:** Predictors of CR at 6 months from univariate logistic regression

Predictor variable	Univariable analysis	
	OR (95% CI)	P value
Proportion of CD19+B cells	1.359 (1.098 to 1.683)	0.005
Proportion of CD3+T cells	0.951 (0.867 to 1.042)	0.279
24-hour urinary protein	0.896 (0.684 to 1.174)	0.426
Serum creatinine	0.990 (0.965 to 1.016)	0.441
Complement C3	0.225 (0.015 to 3.315)	0.277
SLEDAI-2K	1.082 (0.929 to 1.262)	0.311

CR, complete response; SLEDAI-2K, Systemic Lupus Erythematosus Disease Activity Index 2000.

### Adverse events

Three patients reported adverse events (AEs) during follow-up. One patient experienced an upper respiratory tract infection, which resolved by anti-infection therapy. The other two patients presented with mild allergic skin rashes. There were no AEs that required dose reduction or discontinuation of belimumab.

## Discussion

In the present study, we found that add-on belimumab treatment in 28 patients with refractory LN led to significant improvements in proteinuria, hypoalbuminaemia, hypocomplementaemia and disease activity, while renal function remained stable throughout follow-up. Furthermore, both the absolute counts and the proportion of CD19+B cells significantly decreased after 6 months of belimumab treatment. This reduction is consistent with post hoc analysis of the Belimumab International Study in Lupus Nephritis (BLISS-LN) clinical trial that reported similar decreases in CD19+B cells following belimumab therapy.^13^ In contrast, no significant changes were observed in the counts or proportions of CD4+T cells, CD8+T cells and NK cells after treatment.

BLyS is thought to play a role in B cell survival and differentiation, and its dysregulation contributes to the pathogenesis of LN. Elevated BLyS levels in CD19+B cells are correlated with disease activity and autoantibody titres in patients with SLE as well as in those with LN.^14^ In refractory LN, persistent BLyS elevation drives B cell activation and maintains the survival of long-lived plasma cells, which are resistant to conventional therapies.^15^ Accordingly, belimumab has been recommended as first-line treatment for refractory LN in current guidelines.^16 17^ In our cohort, 39.29% of patients achieved CR and 71.43% of patients achieved renal response after 6 months of treatment. This result is similar to a multicentre retrospective study, which showed a renal response rate of 55.6% to belimumab in treating refractory LN at 6 months.^18^ This favourable renal response was accompanied by a significant reduction in CD19+B cells, consistent with belimumab’s mechanism of BLyS inhibition.

Importantly, the baseline proportion of CD19+B cells was significantly higher in patients who achieved CR compared with those who did not, suggesting that CD19+B cell levels may serve as a predictor of treatment response. To further evaluate this predictive value, we performed logistic regression analysis incorporating the proportion of serum CD19+B cells and CD3+T cells, along with four conventional variables, namely 24-hour urinary protein, serum creatinine, complement C3 and SLEDAI-2K, which have been previously identified as independent risk factors for treatment response in LN patients.^11 19 20^ Notably, the baseline proportion of CD19+B cells demonstrated exceptional predictive ability for CR at 6 months. The ROC analysis showed that the proportion of CD19+B cells >15.12% at baseline predicted a 6-month CR with an AUC of 0.888 (95% CI 0.759 to 1.000). This wide CI is a natural consequence of the limited sample size. However, internal validation using 0.632 bootstrap confirmed a stable optimism-corrected AUC of 0.887 with a narrowed 95% CI of 0.835 to 0.924.

An important finding is that a high baseline proportion of CD19+B cells is associated with good treatment response. To the best of our knowledge, there are very few studies that have examined CD19+B cells in predicting renal response to belimumab therapy among refractory LN patients.

This study has several inevitable limitations that need to be acknowledged. First, the sample size was small, partially because of the difficulty in collecting refractory LN patients. As a result, the independent predictive value of baseline CD19+B cell proportion could not be fully evaluated, and the findings should be interpreted as exploratory. Second, this was a single-centre retrospective study. Third, the immunophenotyping data were limited to routine clinical flow cytometry panels. More detailed lymphocyte subsets, such as memory B cells, plasma cells or T cell subsets were not assessed, which may have provided additional mechanistic insights. To overcome these limitations, a prospective multicentre study with a larger sample size incorporating comprehensive lymphocyte subsets analysis is definitely necessary to confirm our findings.

## Conclusion

Our study provides preliminary evidence that the baseline proportion of CD19+B cells may serve as a potential and non-invasive biomarker to predict treatment response to belimumab therapy among refractory LN patients, which may help improve the early treatment decisions for refractory LN patients.

## Supplementary material

10.1136/lupus-2026-002100 online supplemental file 1

## Data Availability

Data are available upon reasonable request.
