# Supplementary material for: Peripheral blood CD19+B cell proportion at baseline predicts early complete response to belimumab treatment in patients with refractory lupus nephritis
Source: Lupus Sci Med. 2026 Aug 3;13(2):e002100. doi: 10.1136/lupus-2026-002100 (PMC13436108; doi:10.1136/lupus-2026-002100)
Supplement: Supplementary file 1 [file lupus-13-2-s001.docx]

**Supplementary material**

**Peripheral blood CD19+ B cell proportion at baseline predicts early complete response to belimumab treatment in patients with refractory lupus nephritis**

Xinxin Xie ^1^, Yan Zhou ^2^, Quangang Zhu ^1*^, Yun Liao ^2*^, Zhongjian Chen ^1*^,

Weihua Song ^2*^

^1^Shanghai Skin Disease Hospital, School of Medicine, Tongji University, Shanghai 200443, China

^2^Department of Clinical Pharmacy, Shanghai General Hospital, Shanghai Jiao Tong University School of Medicine, Shanghai 200080, China

Fig. S1 Change in level of serum CD3+ T cell counts before and after belimumab treatment

Fig. S2 Change in level of serum CD3+ T cells proportion before and after belimumab treatment

Table S1. **Changes in CD4+ T cells, CD8+ T cells, and NK cells** from baseline to 6 months

Table S2. Changes in extra-renal manifestations from baseline to 6 months

Table S3. Exploratory multivariate logistic regression analysis for the prediction of CR at 6 months

Table S4. Internal validation of the predictive model using 0.632 bootstrap with 500 iterations

Table S5. Complete response during the follow-up

Fig. S1 Change in level of serum CD3+ T cell counts before and after belimumab treatment. m, month; ns, no significant.

Fig. S2 Change in level of serum CD3+ T cells proportion before and after belimumab treatment. m, month; ^**^*p*＜0.01.

Table S1. **Changes in CD4+ T cells, CD8+ T cells, and NK cells** from baseline to 6 months

| Cell type | Baseline | 6 months | *p*-value |
| --- | --- | --- | --- |
| CD4+ T cell count (/μL) | 439.0 (250.8, 592.0) | 441.5 (294.0, 697.0) | 0.889 |
| CD4+ T cell proportion (%) | 36.1 ± 8.3 | 37.0 ± 8.5 | 0.699 |
| CD8+ T cell count (/μL) | 493.5 (217.3, 814.3) | 540.5 (322.3, 759.8) | 0.706 |
| CD8+ T cell proportion (%) | 38.1 ± 12.0 | 44.4 ± 12.3 | 0.056 |
| NK cell count (/μL) | 59.0 (29.0, 95.5) | 44.5 (24.3, 115.8) | 0.156 |
| NK cell proportion (%) | 4.8 (3.0, 7.7) | 7.1 (3.7, 13.1) | 0.118 |

Table S2. Changes in extra-renal manifestations baseline to 6 months

| Extra-renal  manifestations | Baseline  n (%) | 6 months  n (%) | *p*-value |
| --- | --- | --- | --- |
| Arthritis | 9 (32.1) | 2 (7.1) | 0.016 |
| Rash | 12 (42.9) | 8 (28.6) | 0.125 |
| Alopecia | 5 (17.9) | 3 (10.7) | 0.625 |
| Thrombocytopenia | 8 (28.6) | 1 (3.6) | 0.039 |
| Leukopenia | 8 (28.6) | 0 (0.0) | 0.008 |

Table S3. Exploratory multivariate logistic regression analysis for the prediction of CR at 6 months

| Predictor variable | Univariable analysis | | Multivariate analysis | |
| --- | --- | --- | --- | --- |
|  | *OR* (95% *CI*) | *p*-value | *OR* (95% *CI*) | *p*-value |
| Proportion of CD19+ B cells | 1.359 (1.098-1.683) | 0.005 | 1.349 (1.092-1.665) | 0.005 |
| 24-hour urine protein | 0.896 (0.684-1.174) | 0.426 | 0.916 (0.626-1.340) | 0.652 |
| Proportion of CD3+ T cells | 0.951 (0.867-1.042) | 0.279 |  |  |
| Serum creatinine | 0.990 (0.965-1.016) | 0.441 |  |  |
| Complement C3 | 0.225 (0.015-3.315) | 0.277 |  |  |
| SLEDAI-2K | 1.082 (0.929-1.262) | 0.311 |  |  |

CR, complete response.

Table S4. Internal validation of the predictive model using 0.632 bootstrap with 500 iterations

| Metric | Original ROC | 0.632 Bootstrap |
| --- | --- | --- |
| AUC | 0.888 | 0.887 |
| 95% *CI* | 0.759-1.000 | 0.835-0.924 |

AUC, area under the ROC curve; *CI*, confidence interval; ROC, Receiver Operating Characteristics.

Table S5. Complete response during the follow-up

|  | 1 month | 3 months | 6 months |
| --- | --- | --- | --- |
| CD19+ B cell proportion >15.12% | 2 | 7 | 10 |
| CD19+ B cell proportion <15.12% | 2 | 2 | 1 |
